# Editorial: Advances in neurodevelopmental and neurodegenerative disease research: focus on innovative human-relevant brain research

**DOI:** 10.3389/fnins.2026.1818513

**Published:** 2026-03-16

**Authors:** Katherine V. Roe, Sara Grassi, Simone Chiola, Richard J. Miller

**Affiliations:** 1 People for the Ethical Treatment of Animals, Norfolk, VA, United States; 2 Dipartimento di Biotecnologie Mediche e Medicina Traslazionale, Universita degli Studi di Milano, Milan, Italy; 3 Stanford University, Stanford, CA, United States; 4 Department of Pharmacology, Northwestern University, Chicago, IL, United States

**Keywords:** Alzheimer's disease, human-relevant research methods, neurodegeneration, neurodevelopment, Parkinson's disease

Neurological and neurodegenerative diseases now represent the leading cause of illness and disability worldwide, placing immense strain on affected individuals, their families, and healthcare systems. Despite this substantial global burden, many of the most prevalent neurodegenerative conditions still have no disease-modifying treatments, and many existing therapies provide only limited symptomatic relief. Although substantial resources have been invested in research aimed at furthering our understanding and treatment neurological disease, progress has been limited in part by the difficulty of studying human brain development directly and by the inability of animal models to capture key human-specific features of brain maturation and disease. Growing recognition of these limitations, together with advances in human iPSC-derived models, neuroimaging, multi-omics technologies, and large human datasets, is transforming the field and enabling more accurate, mechanistically informative, and clinically relevant research.

This Research Topic highlights innovative, human-relevant research spanning Alzheimer's disease (AD), Parkinson's disease (PD), multiple system atrophy (MSA), amyotrophic lateral sclerosis (ALS), spinal muscular atrophy (SMA), and neurodegeneration linked to metabolic dysfunction, bringing together studies that enhance diagnostic accuracy, identify actionable biomarkers, illuminate underlying mechanisms, and advance therapeutic discovery.

Using large-scale epidemiological data, Zhang, Yin et al. examined links between chronic rhinosinusitis (CRS) and Alzheimer's disease risk in the UK Biobank, finding that chronic rhinosinusitis is associated with a modestly increased risk of AD. They also showed that systemic inflammation explains only a tiny fraction of this association, suggesting that other biological pathways may link chronic sinonasal inflammation to neurodegenerative risk.

In a critical study of a previously underrepresented population, Ilie et al. conducted a pilot 16S rRNA sequencing experiment to determine whether individuals with PD show distinct gut microbiome patterns compared to healthy controls. They found that, despite similar overall diversity, PD patients exhibited moderate shifts in microbial community composition, including depletion of several short-chain fatty acid-producing genera, which are often associated with gut and immune health. These results extend gut–brain axis findings to an underrepresented Eastern European population, highlighting regional variability and supporting the growing view that microbiome alterations may play a role in neurodegenerative disease.

While these large-scale studies reveal broad risk patterns, individual cases highlight the nuanced clinical presentations that emerge when these risks manifest in real people. This clinical complexity is illustrated by a case reported by Zhang, Wang et al., who describe a patient whose early symptoms and positive CASPR2 antibodies initially suggested autoimmune encephalopathy, but whose progression, imaging, and electrophysiology data ultimately revealed multiple system atrophy, cerebellar type (MSA-C). The case highlights how antibody findings can mislead diagnosis and shows that careful longitudinal assessment and autonomic testing are essential for distinguishing neurodegenerative disease from treatable autoimmune mimics.

Epidemiological and clinical work defines neurodegenerative disease as it occurs in humans, and other human-relevant approaches are helping clarify the mechanisms involved. For example, Cunningham et al. performed bulk RNA-seq on postmortem caudate nucleus samples from AD and PD patients and identified shared inflammatory and mitochondrial dysfunction signatures, including elevated NFκB1 and NRF2 and reduced GLP-1R, SLC25A6, and HBA1, highlighting convergent molecular pathways across neurodegenerative diseases. Shaikh et al. reviewed evidence that neurotrophic factor alpha 1 (NF-α1) supports neuroprotection, neurogenesis, and synaptic plasticity in AD, and highlighted emerging gene therapy approaches that restore NF-α1 to improve BDNF maturation, reduce amyloid and tau pathology, and enhance cognitive function in AD models. Gao et al. combined multimodal MRI with gut microbiome profiling and found that overweight or obese patients with type 2 diabetes show coordinated changes in appetite-related brain circuits and gut flora, suggesting that disordered ingestive desire arises from linked alterations in brain function, microbial metabolism, and gut–brain signaling.

Alongside these biological investigations, computational methods are now improving how neurodegenerative disease is measured and classified in humans. Sui et al. used a deep-learning MRI segmentation system on clinical scans from 83 PD patients and identified specific cortical and subcortical regions whose volumes track with disease duration, suggesting that AI-derived structural metrics could serve as objective imaging biomarkers of PD progression. Gourdeau et al. applied interpretable machine learning to 38,746 human Montreal Cognitive Assessment (MoCA) evaluations. Their models outperformed the standard fixed MoCA cutoff for identifying cognitive impairment and provided better discrimination among dementia subtypes, including Alzheimer's disease, Lewy body dementia, and frontotemporal dementia.

Another contribution shifts attention to how the broader research landscape is changing. The bibliometric analysis by Sun et al. provides a comprehensive picture of how rapidly research using human induced pluripotent stem cell (hiPSC) models of AD has expanded. Their work shows how central hiPSC-based systems have become for studying human-specific mechanisms of Alzheimer's pathology, and it highlights both the progress achieved and the remaining gaps in this area.

This growing investment in human iPSC-based research is reflected in several studies within this Research Topic. Giadone et al. developed CMAPneuro, a human neuron-based adaptation of the Broad Institute's Connectivity Map platform, differentiating SMA patient-derived iPSCs into NGN2 cortical glutamatergic neurons and exposing them to 360 neuroactive compounds, generating thousands of transcriptional signatures. This approach provides a large, publicly queryable dataset that reveals disease-specific pathways unobservable at baseline and offers a scalable, human-relevant framework for discovering therapeutic candidates. Gomes-Duarte et al. investigated how oxidized phosphatidylcholines (PC-OxPLs) contribute to pathology in SOD1-associated ALS using CRISPR-engineered human iPSC-derived motor neurons carrying the SOD1-G93A mutation. They demonstrated that SOD1-G93A iPSC-derived motor neurons accumulate oxidized phosphatidylcholines and exhibit broad gene expression abnormalities. Neutralizing these lipids with the AAV-delivered intrabody PC-OxPL VecTab^®^ partially restored dysregulated transcripts and prevented axonal damage in culture, positioning PC-OxPL VecTab^®^ as a mechanistically targeted therapeutic candidate and highlighting oxidized lipids as a driver of motor neuron vulnerability.

Taken together, these studies provide an inspiring snapshot of the innovative research tools and evolving research ecosystem that increasingly prioritizes human-relevant models, multimodal data integration, and mechanistic precision, all of which are essential for advancing our understanding of neurodegeneration and accelerating the development of effective therapies.

